# The Aryl Hydrocarbon Receptor (AhR) in the Aging Process: Another Puzzling Role for This Highly Conserved Transcription Factor

**DOI:** 10.3389/fphys.2019.01561

**Published:** 2020-01-14

**Authors:** Vanessa Brinkmann, Niloofar Ale-Agha, Judith Haendeler, Natascia Ventura

**Affiliations:** ^1^IUF – Leibniz Research Institute for Environmental Medicine, Düsseldorf, Germany; ^2^Institute of Clinical Chemistry and Laboratory Diagnostic, Medical Faculty, Heinrich Heine University of Düsseldorf, Düsseldorf, Germany

**Keywords:** aryl hydrocarbon receptor, aging, mitochondria, *C. elegans*, mice, human

## Abstract

Aging is the most important risk factor for the development of major life-threatening diseases such as cardiovascular disorders, cancer, and neurodegenerative disorders. The aging process is characterized by the accumulation of damage to intracellular macromolecules and it is concurrently shaped by genetic, environmental and nutritional factors. These factors influence the functionality of mitochondria, which play a central role in the aging process. Mitochondrial dysfunction is one of the hallmarks of aging and is associated with increased fluxes of ROS leading to damage of mitochondrial components, impaired metabolism of fatty acids, dysregulated glucose metabolism, and damage of adjacent organelles. Interestingly, many of the environmental (e.g., pollutants and other toxicants) and nutritional (e.g., flavonoids, carotenoids) factors influencing aging and mitochondrial function also directly or indirectly affect the activity of a highly conserved transcription factor, the Aryl hydrocarbon Receptor (AhR). Therefore, it is not surprising that many studies have already indicated a role of this versatile transcription factor in the aging process. We also recently found that the AhR promotes aging phenotypes across species. In this manuscript, we systematically review the existing literature on the contradictory studies indicating either pro- or anti-aging effects of the AhR and try to reconcile the seemingly conflicting data considering a possible dependency on the animal model, tissue, as well as level of AhR expression and activation. Moreover, given the crucial role of mitochondria in the aging process, we summarize the growing body of evidence pointing toward the influence of AhR on mitochondria, which can be of potential relevance for aging.

## 1. Aging Hallmarks and Associated Changes

Aging is defined as the time-dependent biological deterioration of structural, cellular and tissue components as well as physiological functions (e.g., stress resistance, immune system, ability to sense and move, decline of organ functionality). It is accompanied by an increased risk for the development of major life-threatening diseases such as cardiovascular disorders, cancer and neurodegenerative disorders (Lopez-Otin et al., 2013; Kaeberlein, 2016). The interest in aging research showed an increase in the past 60 years with a number of almost 7000 new PubMed entries in 2018. 60 years of research have advanced our knowledge on the molecular mechanisms of aging and a number of theories of aging have been proposed (Harman, 1956; Hamilton, 1966; Kirkwood, 1977; Villeponteau, 1997). These theories primarily fall into two categories (i) programmed and (ii) non-programmed based theories. While programmed theories of aging propose that mainly changes in the activity of specific genes, hormones, or the immune system are accountable for the aging process, non-programmed theories consider cellular damage resulting from the interaction with toxicants from the environment or by-products of metabolism the primary factor contributing to aging (Jin, 2010). It seems however most likely that the interaction between genetic and environmental factors shapes the aging process (Kenyon, 2010; Dato et al., 2017). Although aging is a very complex phenomenon and a variety of factors can affect different cells/tissue/organs and their interconnectivity at the same time, different evolutionarily conserved aging hallmarks have been described: (1) genomic instability, (2) telomere attrition, (3) epigenetic alterations, (4) loss of proteostasis, (5) deregulated nutrient sensing, (6) mitochondrial dysfunction, (7) cellular senescence, (8) stem cell exhaustion, and (9) altered intercellular communication (Lopez-Otin et al., 2013; Tigges et al., 2014). Indeed, apart from telomere attrition, all mentioned hallmarks can be observed in vertebrate as well as invertebrate model organisms (Kaeberlein, 2013). Mitochondria clearly play a central role in the aging process and it is interesting to note that while mitochondrial dysfunction itself is one of the hallmarks of aging, severe mitochondrial dysfunction can also promote most if not all other hallmarks of aging.

### 1.1 Mitochondria and Aging

Mitochondria are highly interconnected organelles and are composed of two specialized membranes, an intermembrane space and a matrix containing a circular DNA, which reminds us of their bacterial origin. Mitochondria play a central role in cell and organismal homeostasis and beside their major role in energy metabolism, they also control additional crucial cellular functions ranging from iron and calcium homeostasis to cell death and survival pathways. Given the central importance of mitochondria, cells developed a variety of protective mechanisms to cope with, prevent and repair their damage and alterations thus ensuring cells with the appropriate amount of functional mitochondria in physiological as well as stressful conditions. The “Mitochondrial Free Radical Theory of Aging,” MFRTA, which has taken central stage for several decades (Harman, 1956) in the aging field, states that during life reactive oxygen species (ROS) produced during mitochondria respiration gradually induce irreversible molecular and cellular damages with consequent functional decline ultimately playing a causal role in the aging process. It is therefore not surprising that failure of mitochondrial quality control pathways or severe, non-repairable mitochondrial damage, lead to a plethora of disorders and accelerate the aging process. More surprisingly, yet interestingly, is instead that the MFRTA theory has been recently questioned by the growing body of evidence showing that mild (as opposed to severe) increase in ROS and mild mitochondrial stress can actually promote healthy aging in an evolutionarily conserved manner (Ristow and Zarse, 2010; Ristow and Schmeisser, 2011; Munkacsy and Rea, 2014; Schiavi and Ventura, 2014). This provocative finding (in the field referred to as threshold effect or mitohormesis) has completely changed our classical view of the role of mitochondria in the aging process and stimulated the investigation of novel strategies to promote healthy aging. Taken MFRTA and mitohormesis together it is envisioned that mitochondria play a pivotal role in cell homeostasis and therefore in the aging process.

Dato et al. (2017) estimated that genetic factors only account for one quarter, while environmental and epigenetic factors account for three-quarters of age-associated changes. Considering the high influence of environmental factors on aging, we have recently investigated the role of a central environmental sensor, the highly conserved transcription factor Aryl-hydrocarbon Receptor (AhR) in the aging process (Eckers et al., 2016). Here, we will first review the conflicting evidence pointing to both pro- and anti-aging roles for AhR in aging. We will then try to reconcile these findings based on possible age-, tissue- or dose-dependent activation, and finally discuss pieces of evidence indicating a possible interaction between the AhR and mitochondria, which could be of relevance for the aging process.

## 2. AhR and Aging

The AhR was discovered in 1976 by Poland et al. (1976) as a dioxin-binding protein. The activity of this highly conserved transcription factor is historically dependent on the binding of ligands to its ligand binding domain (LBD). The functionality of this transcription factor is shaped by its functional domains: a basic helix-loop-helix domain (bHLH), two Per-ARNT-Sim (PAS) domains and a transcriptional activation domain (TAD). The N-terminal bHLH domain is involved in DNA binding, binding of heat shock protein 90 (Hsp90), and dimerization with AhR nuclear translocator (Arnt) (Ashida et al., 2008; Abel and Haarmann-Stemmann, 2010). The PAS A domain is required for binding to the Arnt, while the PAS B domain carries the LBD and thus is relevant for ligand binding but also interaction with the AhR-interacting protein [Aip (also XAP2)] and Hsp90. Carboxy-terminal of the AhR is a TAD (Ashida et al., 2008; Abel and Haarmann-Stemmann, 2010). In the absence of ligands, the AhR resides in the cytoplasm bound to Hsp90, Aip, and p23 (Ikuta et al., 1998; Ashida et al., 2008). These co-factors stabilize the ligand-affine state of the AhR and prevent its degradation (Ma and Whitlock, 1997; Meyer and Perdew, 1999). The functions of these co-factors are crucial and AhR is degraded in the absence of Aip or Hsp90 (Hwang et al., 2016). Binding of a ligand causes conformational changes resulting in the exposure of the nuclear localization signal (NLS) and the dissociation from Hsp90, Aip, and p23. In this state, AhR can shuttle to the nucleus, where it dimerizes with Arnt. The AhR-Arnt heterodimer then binds to the xenobiotic responsive elements (XREs) (core sequence 5′-GCGTG-3′) of AhR target genes. These target genes include phase-I detoxification genes like cytochrome P450 (*cyp*) monooxygenase genes (e.g., *cyp1A1* or *cyp1B1*), phase-II detoxification genes like UDP glycosyltransferases (*ugts*) (e.g., *ugt1A1* or *ugt1A6*), and glutathione S-transferases (*gsts*) (e.g., *gstA1* or *gstA2*) (Yueh et al., 2003; Ashida et al., 2008; Xue et al., 2017). To avoid the constant activation of the AhR, negative feedback loops regulate the AhR cascade pathway (Mulero-Navarro and Fernandez-Salguero, 2016; Xue et al., 2017). It is interesting to note that different AhR target genes (e.g., enzymes involved in glutathione synthesis and modulation) as well as genes regulating or regulated by AhR (e.g., Sirt1, p53, Hif1, p300, and HSP90) are involved in the aging process (Henry and Gasiewicz, 1993; Marlowe et al., 2004; Koizumi et al., 2014; Li et al., 2014; Ming et al., 2015; Panchanathan et al., 2015; Ajami et al., 2017; Janssens et al., 2019; Sutter et al., 2019).

Various compounds influence the activity of the AhR, but not all of them are direct ligands. In fact, for some of the compounds modulating the activity of the AhR the direct mechanism is not known. Other compounds modulate AhR activity through an indirect mechanism. For this reason, in this review, we will refer to AhR modulators rather than ligands unless their direct binding to the LBD has been verified. A very well studied group of AhR modulators are xenobiotics. Particularly 2,3,7,8-tetrachlorodibenzo-p-dioxin (dioxin) is a planar molecule that has been shown to bind to the binding pocket of the LBD of mammalian AhR (Poland et al., 1976). Other modulators of the AhR are polyphenols, which can be found in a variety of different fruits and vegetables (Amakura et al., 2003), but whether they really bind to the AhR or affect its activity through other mechanisms is largely elusive (see Xue et al., 2017 for a detailed review). Epigallocatechin gallate, for example, binds to HSP90 and by doing so inhibits AhR signaling (Palermo et al., 2005). Curcumin instead can directly bind the AhR (Ciolino et al., 1998) and inhibits its downstream signaling through inhibition of AhR phosphorylation by the protein kinase C (PKC) (Nishiumi et al., 2007). An *in silico* analysis of the interactions between quercetin and the LBD of human AhR showed that quercetin can bind to specific residues in the AhR binding pocket (Jin et al., 2018). Interestingly, many of the polyphenols modulating AhR activity also affect mitochondria (Sandoval-Acuna et al., 2014). Besides these exogenous modulators, endogenous compounds affect AhR activity as well. These endogenous modulators are mainly, but not solely tryptophan derivatives, like the high-affinity ligand 6-formylindolo[3,2-b]carbazole (FICZ), a photoproduct of tryptophan, which is produced in response to UVB light (Rannug et al., 1987; Fritsche et al., 2007). Another endogenous but low-affinity AhR modulator is kynurenine (Opitz et al., 2011). More recently, compounds produced by the microbiota have been identified as AhR modulators. These are, similarly to the endogenous AhR modulators, mostly derivatives of tryptophan such as indole, indoxyl-3-sulfate, indole-3-propionic acid, indole-3-aldehyde, indole-3-acetate, and tryptamine (Jin et al., 2014; Rothhammer et al., 2016).

It is interesting to note that many of these AhR modulators may affect aging or age-associated diseases. Dioxin exposure, for example, can cause cancer, and cardiovascular diseases (Mandal, 2005; Marinkovic et al., 2010). Exposure to the xenobiotic and AhR ligand benzo[*a*]pyrene, which directly binds to the AhR (Okey et al., 1984) and causes its nuclear localization, shortens the lifespan in mice (Sakakibara et al., 2005), and promotes neurodegeneration as well as Alzheimer’s disease and Parkinson’s disease-like phenotypes in zebrafish (Gao et al., 2015). However, the actual involvement of the AhR in these processes was not investigated in these studies. Instead, in another study, benzo[*a*]pyrene was shown to cause cancer in an AhR-dependent manner (Shimizu et al., 2000) and similarly, the endogenous AhR modulator kynurenine promotes tumor formation through the AhR (Opitz et al., 2011). Moreover, another work showed that mice carrying a low-affinity AhR allele are more susceptible to benzo[*a*]pyrene-induced lethality than mice with a high-affinity AhR allele, suggesting the importance of the degree of AhR activation (Kerley-Hamilton et al., 2012). Many of the plant-derived dietary AhR modulators, on the other hand, have life- and health-extending effects across species. Curcumin, for example, shows protective effects on age-related neurodegenerative diseases in different species (Lim et al., 2001; Alavez et al., 2011; Caesar et al., 2012). Also, the AhR modulator quercetin extends lifespan in *Caenorhabditis elegans (C. elegans)* (Kampkotter et al., 2008; Pietsch et al., 2009), *Drosophila melanogaster* (Drosophila) (Proshkina et al., 2016) and mice (Xu et al., 2018). Interestingly, indole produced by commensal *Escherichia coli* was found to extend the lifespan of *C. elegans*, Drosophila and mice in an AhR-dependent manner (Sonowal et al., 2017) but in all other studies it has not been investigated whether the compounds mediate healthspan in an AhR dependent manner. While all of these studies focus on the effect of specific compounds on aging, there are only a few studies directly linking AhR and aging. In fact, a search on the MEDLINE/PubMed database with the Medical Subject Headings (MeSH) terms “ah receptor” and “aging” gave only 29 results. Here, we want to review the current state of research on the role of the AhR in aging in different model organisms as well as humans. We have however deliberately decided not to describe the large body of association studies correlating AhR activity/expression to age-associated diseases in human, which, although very interesting, would require a separate review.

### 2.1 AhR and Aging in Invertebrates

When studying aging, invertebrate model organisms offer some advantages over vertebrates: they are small, easy to cultivate, cheap in maintenance and most importantly have a short lifespan. These characteristics allow the performance of aging studies with a large number of individuals in a short time. Moreover, the conservation of the major aging pathways, as well as aging features, have made invertebrates like the nematode *C. elegans*, and the fruit fly *Drosophila melanogaster* elected model organisms of aging research (Kenyon, 2010; Kaeberlein, 2013). The homologs of the AhR are *ahr-1* (Powell-Coffman et al., 1998) and *spineless* (Duncan et al., 1998) in *C. elegans* and Drosophila, respectively. AhR in *C. elegans* and Drosophila differ from mammalian AhR in their structure and their ligand binding ability. In fact, the classical AhR ligand dioxin does not bind AHR-1 (Powell-Coffman et al., 1998) and most likely neither Spineless (Duncan et al., 1998). Additionally, no direct binding of a ligand has ever been shown in *C. elegans* or Drosophila. *In vitro* studies showed that the Drosophila AhR is constitutively active which might result in the inability of ligand-dependent activation (Kudo et al., 2009). Recent studies show however that indoles from commensal bacteria extend the lifespan of *C. elegans* and Drosophila in an AhR-dependent manner (Sonowal et al., 2017). This might suggest that microbiota-derived small molecules could act as evolutionarily conserved AhR modulators. For a long time, the main focus of AhR research has been on the response to xenobiotics and the inability of invertebrate AhR to bind dioxin might be one of the reasons why few is known about the function of invertebrate AhRs. However, a conserved function of the AhR in the regulation of developmental processes has been shown in *C. elegans* and Drosophila: *C. elegans ahr-1* mutants develop slightly slower than wild type (Aarnio et al., 2010) and have defects in neuronal development (Huang et al., 2004; Qin and Powell-Coffman, 2004; Qin et al., 2006; Smith et al., 2013). Similarly, Drosophila *spineless* mutants have defects in the development of antenna (Burgess and Duncan, 1990) and sensory neurons (Kim et al., 2006). Another conserved function of the AhR might be the regulation of fertility. *C. elegans ahr-1* mutants have a slightly reduced egg number and an increased embryonic lethality (Aarnio et al., 2010). Although no such function is described in Drosophila, reproduction is affected by AhR in mice (Baba et al., 2008). These studies suggest that the physiological functions of the AhR might be conserved during evolution. Most importantly, AhR has as well an evolutionarily conserved role in the regulation of aging (Eckers et al., 2016). In contrast to the detrimental effects of loss of AhR function in early life, during aging a decreased AhR expression is beneficial: in a cross-species study, we showed that the AhR promotes aging phenotypes in human, mice and *C. elegans* (Eckers et al., 2016) and thus present an evolutionarily conserved role of the AhR in the aging process. More specifically, *C. elegans* carrying a loss of function allele of *ahr-1* had a longer lifespan and an increase in physiological functions (e.g., motility and pharyngeal pumping) and stress resistance during aging (Eckers et al., 2016). Moreover, a higher spontaneous movement activity of *C. elegans*, *D. melanogaster* and humanized mice with reduced *AhR* expression or activity is reported (Williams et al., 2014). Although this is not a direct phenomenon in aging, a decreased movement can be considered a parameter for aging. A potential over-activation of the AhR during the aging process is further supported in *C. elegans* by the observation of increased *ahr-1* mRNA expression during aging (Sonowal et al., 2017).

### 2.2 AhR and Aging in Mice

Currently, four different strains of mice with a complete AhR deficiency (AhR−/−) exist, which have been generated by different laboratories. In two of these strains, the coding part of exon 1 of the AhR gene was replaced with either a neomycin resistance cassette (Fernandez-Salguero et al., 1995) or the bacterial β-galactosidase gene fused to a NLS (Mimura et al., 1997) (thereby deleting the translation start codon as well as a stretch of basic amino acids that may play a role in DNA binding. The third line was generated by deletion of exon 2, which encodes the bHLH domain. Deletion of this exon leads to out of frame splicing from exon 1 into exon 3 and translation termination in codon 24, such that no functional AhR is produced (Schmidt et al., 1996). The AhR-deficiency does not result in lethality during *in utero* development, as the pups in all lines show a Mendelian distribution of the different genotypes (AhR+/+, AhR+/−, AhR−/−). Recently, a fourth AhR knockout mouse model (C57BL/6-*Ahr^TM 1.2Arte^*) was created by Taconic^1^. These mice carry a deletion in exon 3, resulting in an out of frame splicing of exons 2 to exon 4. For simplicity, in this review the four different mice strains will be designated as AhR^Δ1neo/Δ1neo^ (Fernandez-Salguero et al., 1995) AhR^Δ1gal/Δ1gal^ (Mimura et al., 1997), AhR^Δ2/Δ2^ (Schmidt et al., 1996) and AhR^Δ3/Δ3^ (Taconic), respectively. These mouse strains show different phenotypes. On the one hand they exhibit common features like alterations in hepatic development, reproductive health, and retarded growth during the first 4 weeks compared to wild type mice. On the other hand, they show differences in immune system and reaction and susceptibility to infection, which is possibly due, at least in part, to differences in the genetic background (Fernandez-Salguero et al., 1995; Schmidt et al., 1996; Fernandez-Salguero et al., 1997; Lahvis et al., 2005; Baba et al., 2008; Esser, 2009; Butler et al., 2012).

Given that the AhR is heavily involved in detoxification it is not surprising that AhR deficiency has a profound effect on the hepatic system. All four AhR-deficient mice have reduced liver size, portal fibrosis, and a persistent intrahepatic porto-systemic shunt. Furthermore AhR−/− mice display an increased susceptibility to hepatocarcinogenesis and developed larger tumors (Moreno-Marin et al., 2017). In contrast, the AhR^Δ1neo/Δ1neo^ improved the regenerative potential of the lung in response to the deleterious effects of acute toxin exposure (Morales-Hernandez et al., 2017).

AhR is not only involved in xenobiotic metabolism, but also in regulation of inflammation like macrophage M1/M2 polarization and cytokine secretion. It is discussed that AhR activation induces oxidative stress as a result of excessive generation of ROS. Recent studies indicate that AhR also affects several age-associated processes, such as vascular function or cellular senescence and age-associated macular degeneration (Hu et al., 2013; Singh et al., 2014; Eckers et al., 2016; Bravo-Ferrer et al., 2019). The recently generated AhR^Δ3/Δ3^ mice showed a premature aging phenotype resulting in a reduced life span. Those mice display functionality decline in several organs (Fernandez-Salguero et al., 1995; Bravo-Ferrer et al., 2019). In contrast, the AhR^Δ2/Δ2^ mice show a similar survival rate as wild type mice until 15 months of age and do not display a premature aging phenotype (Singh et al., 2014).

Atherosclerosis is assumed as an age-related, chronic inflammatory disease. Several studies have demonstrated that activation of AhR by dioxin or benzo[*a*]pyrene promotes atherosclerosis (Schmidt et al., 1996; Curfs et al., 2005; Wu et al., 2011). AhR overexpressing mice, which display a 10-fold higher affinity to benzo[*a*]pyrene, were crossed to apolipoprotein E deficient mice. Those mice display larger hearts under basal conditions. Moreover, mice showed increased numbers of atherosclerotic plaques in response to benzo[*a*]pyrene compared to a congenic mouse strain expressing an AhR with lower affinity (Kerley-Hamilton et al., 2012). We demonstrated the impact of AhR in the vascular system. By using the AhR^Δ2/Δ2^ mice, which display no phenotype in adulthood, we showed a decrease in vascular stiffness, which was accompanied by increased eNOS-activity and NO-bioavailability (Eckers et al., 2016). On the other hand, AhR^Δ1neo/Δ1neo^ mice demonstrated cardiac hypertrophy, thickening of the arterial media and increased numbers of vascular smooth muscle cells in the arterial wall (Sauzeau et al., 2011).

In summary, the four AhR-deficient mice show different phenotypes with respect to aging (Table 1). However, since the AhR is needed during development and as a response to environmental, one has to consider that an early embryonic defect could result in a different outcome in adulthood. Therefore, it is maybe difficult to use mice, which are total AhR knockouts. One should rather use AhR conditional knockout mice to induce AhR deficiency in adulthood.

**TABLE 1:** Age-related phenotypes in different species and tissues.

**Species**	**Observation**	**AhR regulation^∗^**	**References**
*C. elegans ahr-1(ju145)*	Increased lifespan, movement, heat stress resistance and Pharyngeal pumping	Promotes aging	Eckers et al., 2016
*C. elegans* and *Drosophila melanogaster*	Increased movement	Promotes aging	Williams et al., 2014
*C. elegans ahr-1(ju145) & ahr-1(ia03)*	Increased lifespan	Promotes aging	Sonowal et al., 2017
*Drosophila melanogaster*	Increased heat stress survival	Promotes aging	Sonowal et al., 2017
*Mus musculus* B6.129AhR^Δ1/Δ1G^	Cardiac hypertrophy; macular degeneration; Pyloric hyperplasia; hepatocellular tumors; skin lesions	Prevents aging	Fernandez-Salguero et al., 1995; Fernandez-Salguero et al., 1997
*Mus musculus* B6.129AhR^Δ2/Δ2^	Similar survival rate as wild type mice until 15 months of age	No aging phenotype	Singh et al., 2014
*Mus musculus* B6.129AhR^Δ1/Δ1F^	Bladder cancer in older mice; regress of seminal vesicles	Prevents aging	Baba et al., 2008; Butler et al., 2012
*Mus musculus* AhR^Δ3/Δ3^	Cardiac hypertrophy, liver fibrosis; kyphosis	Prevents aging	Bravo-Ferrer et al., 2019
*Mus musculus* and Human	Positive correlation between macular degeneration and AhR expression	Prevents aging	Hu et al., 2013
*Mus musculus* and Human	Positive correlation between AhR expression and vessel stiffness in the cardiovascular system	Promotes aging	Eckers et al., 2016
Human	Positive correlation between coronary arterial disease and AhR in the cardiovascular system	Promotes aging	Huang et al., 2015

*^∗^Prevents aging: AhR expression/activity prevents aging. Promotes aging: down-regulation of AhR expression/activity promotes aging.*

### 2.3 AhR and Aging in Humans

Evidence for the role of AhR in aging in human subjects or human cell culture systems is rare. Most of these studies focus on the effect of certain AhR modulators on aging or associated parameters. A recent study showed for example that activation of the AhR by airborne polycyclic aromatic hydrocarbons induced cell aging and the expression of aging-related genes in human skin cells (Qiao et al., 2017). Interestingly, the expression of aging-related genes was inhibited by the presence of an AhR antagonist (Qiao et al., 2017). While studies using AhR ligands/modulators are very valuable for finding treatments for the prevention of pollution-induced aging or disease phenotypes, the direct effect of AhR in these processes is elusive since even high-affinity ligands like dioxin also affect cells in an AhR-independent manner (Hossain et al., 1998). There are only very few studies on the role of AhR on aging in the absence of modulators. A study from 2013 shows that AhR activity decreases during aging in human retinal pigment epithelial cells (Hu et al., 2013). Moreover, AhR protein levels were lower in cells from old donors compared to young donors, while AhR mRNA levels remained unaltered (Hu et al., 2013). They verified their findings in a mouse model and associated decreased activity of the AhR to age-related macular degeneration-like pathology (Hu et al., 2013). In 2016 we found that AhR expression is positively correlated to cardiovascular aging in humans (Eckers et al., 2016). Pulse wave velocity as the up to now best marker for vascular aging is increased with age and is positively correlated with AhR mRNA levels. Thus, increased AhR expression seems to be associated with old age in humans, thus we propose that AhR expression level is an indicator for vessel functionality (Eckers et al., 2016). Along the same lines, AhR expression has been linked to the incidence of coronary arterial disease in an epidemiological study on a Chinese population (Huang et al., 2015). They found increased AhR mRNA expression in coronary arterial disease patients compared to controls and suggested AhR as a diagnostic biomarker for coronary arterial disease (Huang et al., 2015).

In summary, the few studies on the role of AhR in human aging, similar to mice studies, display a complex role of the AhR. It has to be noted that one should clearly separate the effects of AhR in the development and in the aging process from invertebrates to vertebrates. However, it could be possible that the effect of the AhR on aging is tissue-dependent (Table 1) as well as environment dependent. Thus, more research in adult and aged invertebrates, vertebrates and humans is needed to understand the pathophysiological role of AhR in aging in different tissues, organs, as well as in the whole organism.

## 3. AhR-Mitochondria Crosstalk

Mitochondria play a central role in the aging process, are targeted by environmental pollutants and represent a central hub in nutrient metabolism. Interestingly, both environmental pollutants and dietary factors, such as polyphenols, can influence the transcriptional activity of the AhR. We, therefore, envisioned a possible crosstalk between AhR and mitochondria. Surprisingly, while mitochondria are extensively studied (more than 150,000 publications on PubMed) and, there are many studies investigating the influence of different AhR modulators on mitochondria, there are only 32 publications directly linking mitochondria to the AhR.

The effects of polyphenols on AhR and mitochondria are complex (reviewed in Sandoval-Acuna et al., 2014; Xue et al., 2017). On the one hand natural polyphenols are ROS scavengers and thereby influence mitochondria, which are targeted by ROS, but also mitochondrial down-stream signaling through the scavenging of mitochondrial ROS. This ROS scavenging function might be as well important for the influence of polyphenols on AhR activity. ROS can activate AhR through the conversion of tryptophan to FICZ (Smirnova et al., 2016) and thus the ROS-scavenging properties of polyphenols might prevent this activation and connect AhR with mitochondria. On the other hand, additional ROS-scavenging independent functions of polyphenols are reported on mitochondria like regulation of mitochondrial biogenesis, mitochondrial membrane potential, and mitochondrial electron transport chain activity (Sandoval-Acuna et al., 2014). Given the central role of Sirt1 in the aging process, a very interesting ROS-scavenging independent mode of action of polyphenols on mitochondria is the induction of mitochondrial biogenesis through Sirt1. Several polyphenols indeed were shown to activate Sirt1 (reviewed in Ajami et al., 2017). Considering the interaction between AhR and Sirt1 (Koizumi et al., 2014; Ming et al., 2015; Sutter et al., 2019), the notion that ROS activates AhR (Smirnova et al., 2016), and the multiple roles polyphenols may exert on mitochondria, these findings provide indirect evidence for a possible crosstalk between AhR, Sirt1 and mitochondria of relevance for the aging process.

The first direct evidence of a link between mitochondria and the AhR was published in 2002 (Senft et al., 2002). In this study, Senft and co-workers investigated the role of AhR signaling in the increase of mitochondrial ROS upon dioxin treatment in the liver of mice. Specifically, they found that dioxin treatment induced mitochondrial ROS in wild type but not in AhR−/− mice (Senft et al., 2002). Interestingly, they noticed that the basal mitochondrial ROS levels were lower in AhR−/− mice (Senft et al., 2002), which might suggest that AhR has an impact on the mitochondria not only in the presence of ligands but also under normal conditions. Dioxin exposure was also shown to decrease mitochondrial membrane potential in spermatozoa of mice in an AhR-dependent manner (Fisher et al., 2005). Similarly, embryonic stem cells and beating cardiomyocytes from AhR−/− mice are protected against the dioxin-induced increase in markers of mitochondrial stress and of mtDNA damage (Wang et al., 2016). Together, these studies suggest that AhR mediates mitochondrial dysfunction in response to dioxin. Moreover, benzo[*a*]pyrene was shown to increase mitochondrial dysfunction and decrease the mitochondrial membrane potential, resulting in the depletion of ATP levels along with inhibition of the oxygen consumption rate in the human keratinocyte cell line (HACAT). In this study, it was shown that the removal of damaged mitochondria by mitophagy is reduced in AhR and CYP1B1 (an AhR target gene) knockdown but a direct link between AhR and mitophagy was not established (Das et al., 2017). In another study genetic ablation of the AhR resulted in reduced expression of Superoxide Dismutases (SODs) in fibroblasts. Thus, those fibroblasts are more sensitive to cigarette smoke resulting in increased cell death and reduced proliferation, which is accompanied by decreased mitochondrial membrane potential (Rico De Souza et al., 2011). The detrimental effect of loss of AhR function is further supported by studies in embryonic hearts of mice, where the disruption of AhR signaling leads to mitochondrial dysfunction (Carreira et al., 2015b). Similarly, female, but not male mice exposed to dioxin as embryos showed altered expression in genes of the canonical mitochondrial pathway and a higher number of mitochondria in the heart (Carreira et al., 2015a). In adult AhR−/− mice those changes were not observed (Carreira et al., 2015a). In both studies the effect of dioxin treatment was not investigated in AhR−/− mice.

AhR does not only influence mitochondrial function but two studies have recently suggested that the AhR is also localized within the mitochondria. Tappenden et al. (2011) were the first to identify an interaction between the AhR and the ATP5α1 subunit of the ATP synthase complex in different cell lines. Further analysis of the exact localization of the mitochondrial AhR in murine hepatoma cells showed that it localizes inside the intermembrane space (Hwang et al., 2016). Interestingly, when treated with dioxin, mitochondrial localization of the AhR and interaction with ATP5α1 were lost (Tappenden et al., 2011; Hwang et al., 2016), suggesting that AhR only localizes inside the mitochondria in the absence of ligands. Considering that AhR is bound by AIP in the absence of ligands and that AIP has been found to interact with the Mitochondrial import receptor subunit TOMM20 and to mediate preprotein transport in mitochondria (Yano et al., 2003), AIP could be the critical mediator of AhR localization into mitochondria. Indeed, siRNA against TOMM20 reduces mitochondrial AhR, but not cytoplasmatic or nuclear AhR by 70% (Hwang et al., 2016). Thus, Hwang et al. proposed that AIP and HSP90 contribute to the mitochondrial localization of AhR by interacting with TOMM20, which imports AhR into the intermembrane space (Hwang et al., 2016).

Taken together, these studies strongly suggest that the effects of AhR on mitochondrial function are likely tissue-, age-, and maybe even sex-dependent (Table 2). Additionally, the presence of dioxin seems to have a strong impact on the outcome of the study. Nonetheless, these are mainly *in vitro* studies and causal-effect, as well as mechanistic studies in primary cells and model organisms in more physiological conditions, are required to clearly establish a possible crosstalk between AhR and mitochondria. Interestingly, in *C. elegans*, animals with reduced mitochondrial function and *ahr-1* mutants share some phenotypic features like slower larval development, alterations in fat metabolism and sensory neurons and most importantly lifespan extension (Rea et al., 2007; Aarnio et al., 2010; Schiavi et al., 2013; Smith et al., 2013; Maglioni et al., 2014; Eckers et al., 2016). Moreover, long-lived mitochondrial mutants have increased levels of *cyps*, *ugts*, and *gsts* (Cristina et al., 2009; Liu et al., 2014; Mao et al., 2019), which, at least in mammals are known target genes of the AhR. Investigating the potential AhR-mitochondrial crosstalk in appropriate *in vivo* model systems will certainly help revealing its potential causal role in different pathophysiological contexts including aging and associated pathologies.

**TABLE 2:** Tissue- and age-specific effects of AhR expression on mitochondrial function in mice.

**Tissue/cells**	**Age**	**Observation**	**Effect of AhR−/−**	**References**
Liver		Dioxin exposure increases mitochondrial ROS	Protective to dioxin exposure	Senft et al., 2002
Spermatozoa		Dioxin exposure decreases the mitochondrial membrane potential	Protective to dioxin exposure	Fisher et al., 2005
Fibroblasts		Reduced expression of SODs in AhR deficient cells	Detrimental to cigarette smoke	Rico De Souza et al., 2011
Heart	Embryo	AhR−/− induce mitochondrial dysfunction	Detrimental	Carreira et al., 2015b
	Adult	Dioxin treatment of embryos induces mitochondrial dysfunction in adults	No effect	Carreira et al., 2015a

## Author Contributions

NV and JH contributed to the conception of the review. VB and NA-A wrote the sections of the manuscript. All authors contributed to the manuscript revision, editing read, and approved the submitted version.

## Conflict of Interest

The authors declare that the research was conducted in the absence of any commercial or financial relationships that could be construed as a potential conflict of interest.

## Footnotes

1 https://www.taconic.com/mouse-model/ahr-knockout-mouse
